# Extensive influence of microsporidian infection on sucrose solution consumption, antioxidant enzyme activity, cell structure, and lifespan of Asian honeybees

**DOI:** 10.3389/fimmu.2024.1404766

**Published:** 2024-11-19

**Authors:** Xiaoxue Fan, Haodong Zhao, He Zang, Shunan Dong, Jianfeng Qiu, Yuxuan Song, Kunze Li, Haibin Jiang, Ying Wu, Yang Lü, Dingding Zhou, Zhongmin Fu, Dafu Chen, Rui Guo

**Affiliations:** ^1^ College of Bee Science and Biomedicine, Fujian Agriculture and Forestry University, Fuzhou, Fujian, China; ^2^ National & Local United Engineering Laboratory of Natural Biotoxin, Fujian Agriculture and Forestry University, Fuzhou, Fujian, China; ^3^ Apitherapy Research Institute of Fujian Agriculture and Forestry University, Fuzhou, Fujian, China; ^4^ Bee Pollination and Product Safety Research Laboratory, Apiculture Science Institute of Jilin Province, Jilin, Jilin, China; ^5^ Bee Research Institute, Heilongjiang Academy of Agricultural Sciences, Mudanjiang, Heilongjiang, China

**Keywords:** honeybee, *Apis cerana*, *Vairimorpha (Nosema) ceranae*, host-parasite interaction, antioxidant enzyme

## Abstract

*Apis cerana* is the original host of *Vairimorpha (Nosema)* ceranae, a widespread fungal parasite that causes bee nosemosis, which severely threatens the health of bee colonies and the sustainable development of the apiculture industry. To evaluate the impact of *V. ceranae* infection on *A. c. cerana* workers, *V. ceranae* spores were purified and used to inoculate newly emerged workers to evaluate the effects of *V. ceranae* infection. This was followed by an in-depth investigation of *V. ceranae* spore load and host sucrose solution consumption. Activities of four major antioxidant enzymes (SOD, PPO, CAT, and GST) were determined. Paraffin sections of the host midgut tissue were prepared and subjected to microscopic observation. The survival rates of *V. ceranae*-inoculated and uninoculated workers were analyzed. The results showed that spore load gradually increased and peaked at 12 dpi. The consumption of workers in the *V. ceranae*-inoculated group was extremely significant higher (*P* < 0.0001) than that of workers in the un-inoculated group. The results of antioxidant enzyme activity were suggestive of positive host defense via catalase (CAT) and glutathione-S-transferase (GST) in the middle stage of infection, as well as the negative fungal impact on superoxide dismutase (SOD) and polyphenol oxidase (PPO) at the whole stage of infection, reflecting the complex host-parasite interaction. Additionally, we observed a disruption in the structure of the host midgut epithelial cells. Moreover, the survival rate of workers in *V. ceranae*-inoculated groups was nearly always lower than that of workers in the uninoculated groups. These results demonstrate a consistent increase in spore load with the proliferation of *V. ceranae*, leading to persistent energetic stress and midgut epithelial cell structural damage to the host, ultimately resulting in a shortened lifespan for the host. Our findings enhance the current understanding of the interactions between *A. cerana* and *V. ceranae* as well as provide a solid basis for exploring the mechanisms underlying host response and *V. ceranae* infection.

## 1 Introduction

*Vairimorpha* (*Nosema*) *ceranae* is a fungal parasite that causes nosemosis, a chronic disease that is frequently observed in bee colonies worldwide (1). In 1996, Fries et al. (2). first identified *V. ceranae* in *A. cerana* colonies, and established *A. cerana* as the original host of this parasite. Since its initial discovery in Europe in 2006, *V. ceranae* has rapidly spread to other parts of the world, North America, South America, Asia, Africa, and likely other regions as well, likely facilitated by the global trade of bees, bee products, and the movement of beekeeping equipment (3).

*V. ceranae* is associated with a decline in bee health, reduced colony productivity, and colony collapse disorders (CCD) (4, 5). Over the past two decades, a substantial number of studies have been conducted to investigate the effect of *V. ceranae* infection on *Apis mellifera*, demonstrating that fungal invasion negatively influences various aspects of honeybee metabolism, behavior, physiology, and health (6–12). For instance, studies have shown that the *V. ceranae* infection cause metabolic stress, upregulates genes encoding α-glucosidase and trehalose transport enzymes (9), while downregulating trehalase and Glucose-Methanol-Choline oxidoreductase genes (11). These alterations, as confirmed by proteomics, resulted in reduced levels of energy supply proteins (8). The behavioral consequences of *V. ceranae* infection in *A. mellifera* are also noteworthy, as infected bees often display increased hunger and consume more sucrose to compensate for their impaired metabolic state. However, this increased food intake does not fully alleviate the underlying nutritional and energy deficits (6), and accelerated lipid loss in bees suggests that lipids may be mobilized as an emergency energy source (12). *V. ceranae* infection can damage the midgut epithelial cell structure, inhibit apoptosis and immunosuppression, and shorten the lifespan of *A. mellifera* workers (7, 10).

Through extensive interactions and long-term co-evolution, *A. cerana* and *V. ceranae* have evolved to coexist (13). Previous studies have investigated the effects of *V. ceranae* infections on *A. cerana* (14–17). Sinpoo et al. (14) found stronger immune responses and lower loads of *Nosema apis* and *V. ceranae* spores in workers of *A. cerana* compared to *A. mellifera*, suggesting a higher tolerance of *A. cerana* against microsporidian infection. Chantaphanwattana et al. (18) reported that *V. ceranae* proliferated more in *A. mellifera*, whereas *A. cerana* exhibited a stronger immune response and lower spore load, indicating a superior defense mechanism of *A. cerana* at the individual level. By contrasting the similarities and differences in *V. ceranae* infection of *A. cerana* and *A. mellifera*, a deeper understanding of the complex interactions between *V. ceranae* and bee hosts, as well as the factors responsible for coaptation between *A. cerana* and *V. ceranae* will be gained. In addition, novel and valuable insights from the aforementioned comparisons could guide the creation of targeted strategies to alleviate the adverse effects of fungal parasites on different bee species.

*A. c. cerana* is a bee species that is found in China and is widely used in beekeeping practice in several Asian countries (19). Previously, our team identified the miRNA response and miRNA-regulated network of *A. c. cerana* workers following *V. ceranae* invasion (20), and performed a comprehensive investigation of the profiles of highly expressed genes in both the host and microsporidian (21, 22). Recently, our group investigated the immune response of *A. c. cerana* workers to *V. ceranae* infection using a transcriptomic investigation and revealed that different cellular and humoral immune responses were used by *A. c. cerana* and *A. mellifera ligustica* workers to defend themselves against infection by *V. Ceranae* (23). Moreover, this study provides an opportunity for a deeper comparison of the effect of the same fungal parasite (*V. ceranae*) on two different bee species (*A. cerana* and *A. mellifera*), which will enrich our understanding of bee nosemosis and facilitate the development of biological drugs for nosemosis control.

Many aspects of the effect of *V. ceranae* on *A. cerana* are yet to be explored. Pathogenic infections in insects result in the generation of reactive oxygen species (ROS) that can kill and eliminate pathogens (24). However, excessive production of ROS *in vivo* induces oxidative stress, predominantly damaging various biological macromolecules, and ultimately resulting in cell death (25). Antioxidant enzymes reduce the oxidative response to balance ROS production and maintain homeostasis (26). Superoxide dismutase (SOD) catalyzes the disproportionation of superoxide to produce hydrogen peroxide and molecular oxygen, whereas catalase (CAT) participates in the decomposition of hydrogen peroxide into water and oxygen (27–29). Glutathione-S-transferase (GST) has been shown to exert function in metabolizing lipid peroxides (27, 30). Polyphenol oxidase (PPO) is an oxidoreductase that uses molecular oxygen to catalyze the oxidation of a wide range of phenolic compounds (31). Insects appear to use PPO in immune responses such as melanization to physically limit the spread of microbes (32). Whether *V. ceranae* infection affects the activities of major antioxidant enzymes in *A. cerana* remains largely unknown.

Currently, little is known regarding the effect of *V. ceranae* infection on *A. cerana*, hindering the understanding of the mechanisms underlying the interaction between *V. ceranae* and *A. cerana* and the development of novel strategies for the control of bee nosemosis. Here, to evaluate the impact of *V. ceranae* infection on *A. c. cerana* workers, *V. ceranae* spores were purified and used to inoculate newly emerged workers to evaluate the effects of *V. ceranae* infection. This was followed by an in-depth investigation of *V. ceranae* spore load and host sucrose solution consumption. Activities of four major antioxidant enzymes (SOD, PPO, CAT, and GST) were determined. Paraffin sections of the host midgut tissue were prepared and subjected to microscopic observation. The survival rates of *V. ceranae*-inoculated and uninoculated workers were analyzed. The findings from this study provide valuable experimental evidence for *V. ceranae* infection of *A. c. cerana* workers and contribute to the dissection of the host response, microsporidian infection, and host–microsporidian interaction.

## 2 Materials and methods

### 2.1 Spore extraction, artificial inoculation, and honeybee preparation for bioassay experiments

*A. c. cerana* workers were obtained from colonies in the teaching apiary of the College of Bee Science and Biomedicine, Fujian Agricultural and Forestry University, in October 2020. There were no specific bands amplified from *V. apis*, *V. ceranae*, or several viruses, such as SBV (Sacbrood Virus), DWV (Deformed Wing Virus), IAPV (Israeli Acute Paralysis Virus), BQCV (Black Queen Cell Virus), KBV (Kashmir Bee Virus), and CBPV (Chronic Bee Paralysis Virus) based on reverse transcription-polymerase chain reaction (RT-PCR) with specific corresponding primers, that eliminated the interference of pathogens on the samples.

*V. ceranae*-infected *A. c. cerana* workers were obtained from an apiary in Minhou, Fuzhou, China. *V. ceranae* spores were prepared using the discontinuous density gradient Percoll method, as described by Chen et al. (33). Briefly, (1) a total of 200 foragers were collected from an infected colony, the midgut tissues were dissected and then homogenized in distilled water, followed by filtration through four layers of sterile gauze and three cycles of centrifugation at 6000 × g for 5 min; (2) the supernatant was discarded as the spores remained in the sediment, and the resuspended pellet was then purified on a discontinuous Percoll gradient (Solarbio) consisting of 200 μL each of 25%, 50%, 75%, and 100% Percoll solution; (3) the spore suspension was overlaid onto the gradient and centrifuged at 18000 × g for 90 min at 4°C; (4) the spore pellet was isolated using a sterile syringe and then centrifuged again on a discontinuous Percoll gradient. Purified spores were examined by PCR using previously reported primers (34) and then preserved in our laboratory and at the China Common Microbial Strain Preservation and Management Center (Preservation number CGMCC: No. 28110). The spore concentration was determined using a CL kurt counter for the inoculation experiments.

Before artificial inoculation, the prepared microsporidian spores were observed microscopically and examined using PCR with specific primers for *V. ceranae* (F: 5′-CGGATAAAAGAGTCCGTTACC-3′; R: 5′-TGAGCAGGGTTCTAGGGAT-3′) (17, 20) and *V. apis* (F: 5′-CCATTGCCGGATAAGAGAGT-3′; R: 5′-CACGCATTGCTGCATCATTGAC-3′), as previously described by Chen et al. (34). The inoculation and rearing of *A. c. cerana* workers were performed according to a previously established protocol (20). At 24 h after emergence, members of the treatment group (*n* = 35) were each immobilized and fed 5 μL of 50% (w/v) sucrose solution containing 1 × 10^6^ V*. ceranae* spores by using a pipette, whereas members of the control group (*n* = 35) were fed 5 μL of sucrose solution without *V. ceranae* spores. The treatment (or control) group contained six plastic cages. All workers were reared in an incubator at 34 ± 0.5°C and 60–70% RH (relative humidity). After 24 h, the honeybees were fed a feeder containing 4 mL of 50% (w/v) sucrose solution, and the feeders were replaced daily throughout the experiment. Each cage was carefully checked, and dead honeybees were removed daily. Three biological replicates were used in each experiment.

The prepared workers from *V. ceranae*- and uninoculated groups underwent spore load measurement (*n* = 3), antioxidant enzyme activity examination (*n* = 3), hematoxylin and eosin staining (HE) of paraffin sections (*n* = 3).

### 2.2 Measurement of spore load in midguts of the workers

During the *V. ceranae* infection process, 1–12 days post inoculation (dpi), the spores in the midguts of the workers were extracted every 24 h based on the standard method described by Fries et al. (35) and Naree et al. (16), followed by calculation under an optical microscope (CSOIF, Shanghai, China), according to the procedure described by Cantwell (36). In brief, (1) after inoculation with *V. ceranae*, a single worker in the treatment group was collected from the cage daily, and their midgut tissues were then dissected using a clean ophthalmic forced and transferred into a sterile 1.5-mL Eppendorf (EP) tube; (2) 100 μL of sterile water was added to the EP tube, and then full grinding with an automatic high throughput tissue grinder (Meibi, Zhejiang, China) was performed; (3) using a micropipette, 20 μL of the aforementioned solution was carefully observed with a hemocytometer plate (Qiujing, Shanghai, China) under an optical microscope (CSOIF, Shanghai, China), after which spores were counted.

### 2.3 Detection of host average sucrose solution consumption

According to the method described in Section 2.1, separate experimental workers were prepared for the purpose of statistical analysis of sucrose solution consumption and survival rates. The daily average consumption of sucrose solution by each worker in the *V. ceranae*- and uninoculated groups was analyzed using a previously described method (37, 38). The feeder containing 4 mL of sucrose solution was weighed and the weight was recorded as A. To avoid errors due to repeated changes in feeders, the feeder was weighed every 24 h thereafter and the weight was recorded as B. The sucrose solution in the feeders was replaced daily. The numbers of survivors in both *V. ceranae*- and un-inoculated groups were counted. The average sucrose solution consumption per worker per day was calculated according to the following formula: (B-A)/number of survivors in the *V. ceranae*-inoculated group (or uninoculated group). Each biological replicate was performed three times, *n* = 35.

### 2.4 Examination of the antioxidant enzyme activity

At 1–12 dpi, three workers were randomly selected from *V. ceranae*- and un-inoculated groups, the midgut tissues were carefully dissected and then added into a sterile EP tube containing 180 µL of saline followed by fully ground with a high-throughput tissue grinder (MEIBI, Hangzhou, China), respectively. The grinding fluid was centrifugation at 5000 × g for 5 min, and the supernatant was then transferred to a sterile EP tube, stored at −80°C. Following the corresponding instructions, the activities of SOD and PPO were examined using the Insect SOD ELISA Kit (MLBIO, Shanghai, China) and Insect PPO ELISA Kit (MLBIO, Shanghai, China), respectively, whereas those of CAT and GST were determined using the Insect CAT ELISA Kit (MLBIO, Shanghai, China) and Insect GST ELISA Kit (MLBIO, Shanghai, China), respectively. The experiment included three biological replicas. Specific antioxidant enzyme activity was expressed as units of enzyme activity per milligram of protein.

### 2.5 Paraffin sectioning, HE staining, and microscopic observation of midgut tissues of workers

At 7, 8, 9, and 10 dpi, three workers were randomly selected from *V. ceranae*- and uninoculated groups, and the midgut tissues were carefully dissected. Next, according to the method described by Xing et al. (23), midgut tissue from one of the workers was fixed with 4% paraformaldehyde (PFA). By using an embedding center (Junjie, Wuhan, China) and a microtome (Leica, Nussloch, Germany), paraffin sections of midgut tissues were stained with HE staining by Shanghai Sangon Biological Engineering Co. Ltd., and then detected under an optical microscope with digital camera (SOPTOP, Shanghai, China).

### 2.6 Survival rate of workers

Using the same experimental group as in Section 2.3, before replacing the sucrose solution every 24 hours, the survival status of workers in both the *V. ceranae*-inoculated group and the uninoculated group was recorded, and dead ones were removed. This recording was continued until 20 dpi. Subsequently, the daily survival rates for the both groups were calculated on basis of the formula: (number of living worker bees in the treatment or control group)/105.

### 2.7 Statistics

Statistical analysis of the data was performed using SPSS software. 21 (IBM, Armonk, NY, USA) and GraphPad Prism 6.0 software (GraphPad, San Diego, CA, USA). To analyze significant differences among multiple groups of data, either One-way ANOVA (followed by Tukey’s *post-hoc* test), Two-way ANOVA (followed by Bonferroni’s *post-hoc* test), multiple t tests was performed on the spore load and average sucrose solution consumption, and activity of SOD, PPO, GST and CAT using the SPSS software. Data are presented as the mean ± standard deviation (SD). Tukey’s test and the significant difference letter marker method were used to compare the number of spore loads between the 2 d. The log-rank (Mantel–Cox) test was used to analyze the host survival rate.

## 3 Results

### 3.1 Dynamics of *V. ceranae* spore load in the midguts of *A. c. cerana* workers

**Figure 1:**
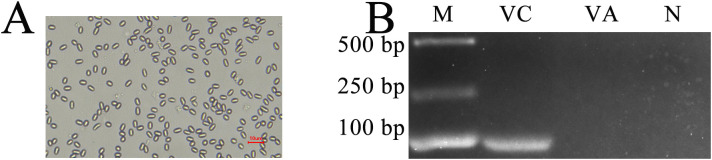
**(A)** Microscopic observation of *V. ceranae* spores derived from Percoll discontinuous density centrifugation (400× amplification). **(B)** Agarose gel electrophoresis for PCR amplification products from purified spores. Lane M: DNA marker; Lane VC: specific primers for *V. ceranae*; Lane VA: specific primers for *V. apis*; Lane N: sterile water (negative control).

After discontinuous Percoll gradient centrifugation, oval spores with high refraction were observed using an optical microscope (OM) (**Figure 1A**). In addition, the expected fragment (76 bp) could be amplified from the purified spores with specific primers for *V. ceranae* but could not be amplified from sterile water and spores with *V. apis* specific primers (**Figure 1B**). These results confirmed that the prepared spores were *V. ceranae* spores.

**Figure 2:**
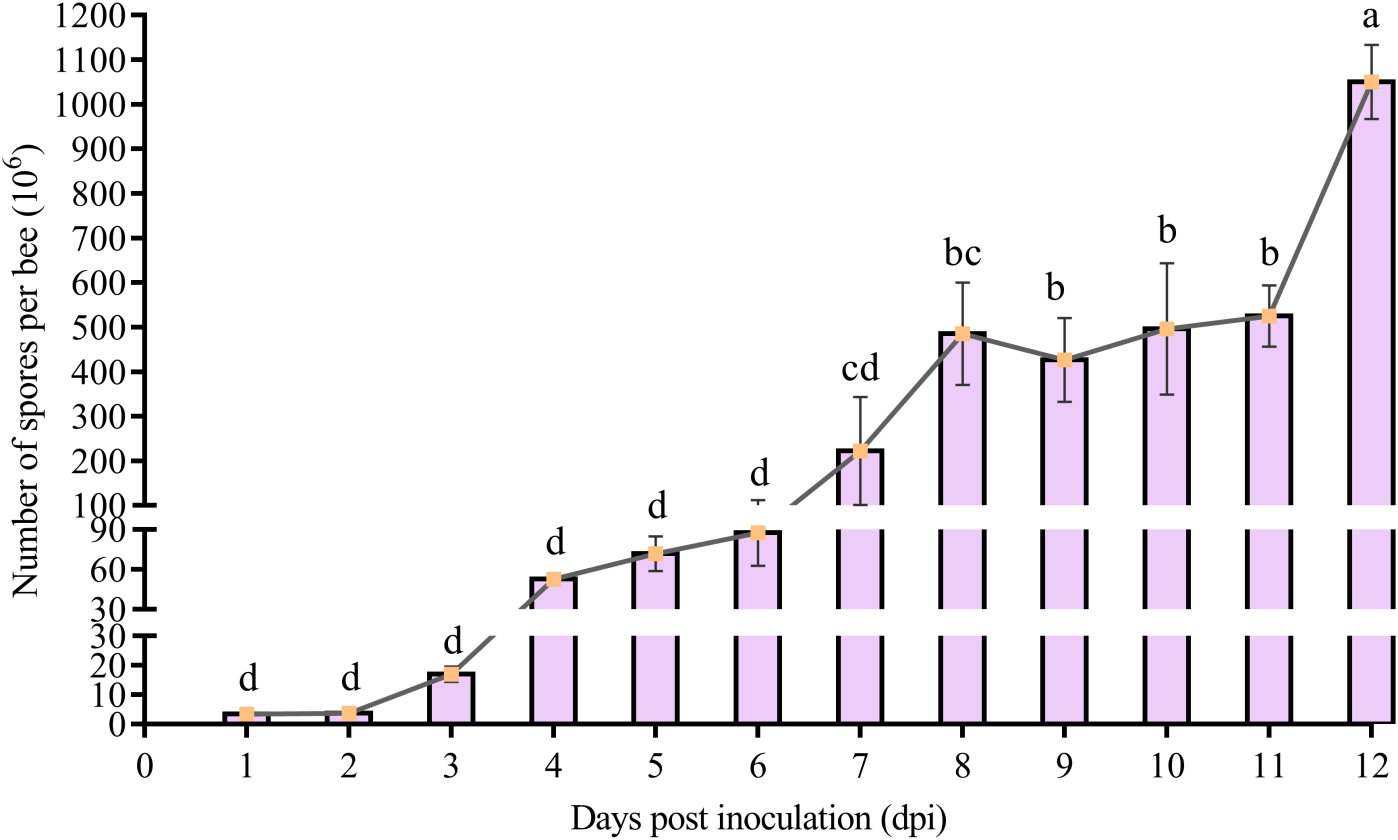
Spore load of *V. ceranae* in the midguts of *A. c. cerana* workers after inoculation (*n*=3). Whiskers indicate the mean ± SD. Data were analyzed using the One-way ANOVA (Tukey’s *post-hoc* test). The same lowercase letters above the curve indicate no significant difference (*P* > 0.05), whereas different lowercase letters above the curve indicate statistically significant differences (*P* < 0.05).

The spore counting results suggested that the spore load of *V. ceranae* in the midguts of workers presented an overall elevation trend (**Figure 2**). In detail, as compared with initial spore load (1 × 10^6^), the *V. ceranae* spore load in the midguts of the workers continuously increased from 1 dpi (3.5 ± 0.87 × 10^6^) to 4 dpi (52.83 ± 2.75 × 10^6^), from 5 dpi (72 ± 13.08 × 10^6^) to 8 dpi (48.53 ± 11.533 × 10^7^), and from 9 dpi (42.68 ± 9.42 × 10^7^) to 12 dpi (10.51 ± 0.83 × 10^8^) (**Figure 2**); however, the spore load was decreased from 8 to 9 dpi. In addition, the quantity of spores reached the peak at 12 dpi (**Figure 2**).

### 3.2 Influence of *V. ceranae* infection on average sucrose solution consumption of *A. c. cerana* workers

**Figure 3:**
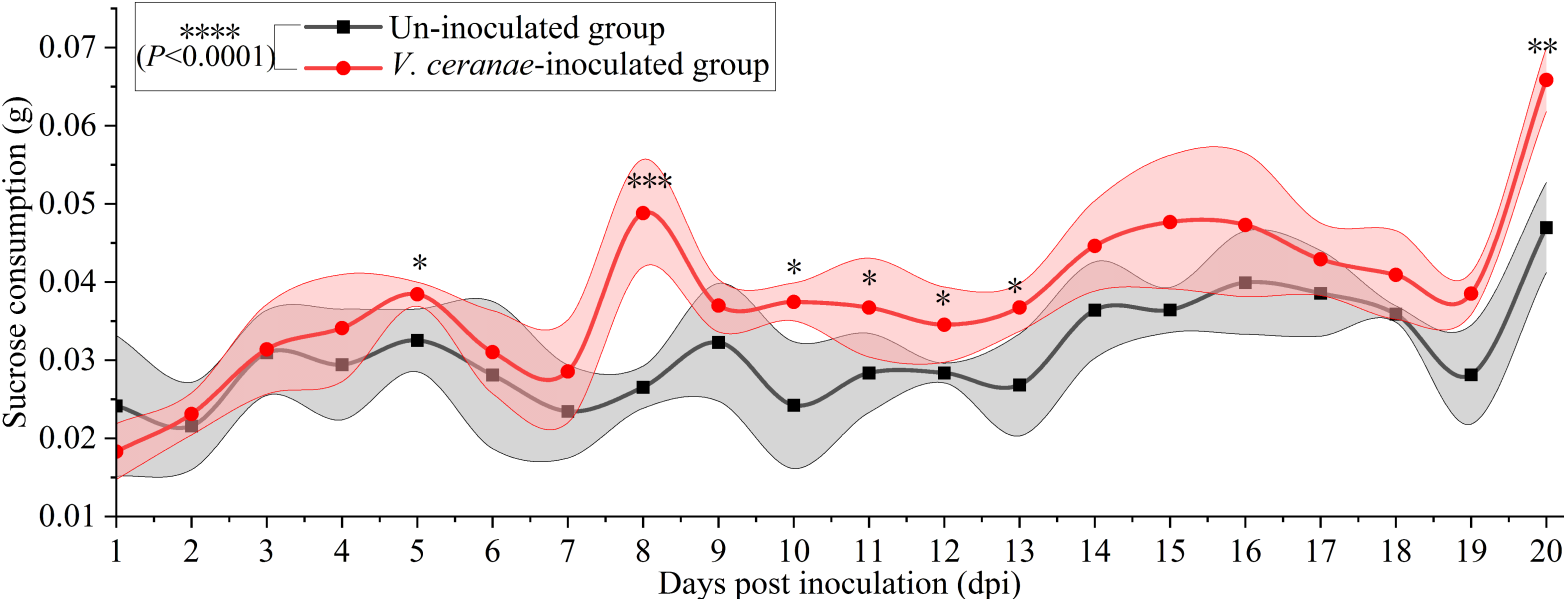
Average sucrose solution consumption of *V. ceranae*-inoculated and un-inoculated workers (*n*=105). Data were showed as mean ± SD, that analyzed using the Two-way ANOVA (Bonferroni’s *post-hoc* test), multiple t tests; * indicates *P* < 0.05, ** indicates *P* < 0.01, *** indicates *P* < 0.001, **** indicates *P* < 0.0001.

The average sucrose consumption of *V. ceranae*-inoculated and un-inoculated workers was 0.0308 ± 0.0080 g/d and 0.0371 ± 0.0107 g/d, respectively. The consumption of workers in the *V. ceranae*-inoculated group was extremely significant higher (*P*<0.0001) than that of workers in the un-inoculated group, significant difference in the average sucrose solution consumption between the two groups was observed at 5 (*P* = 0.0244), 8 dpi (*P* = 0.0005), 10 dpi (*P* = 0.0443), 11 dpi (*P* = 0.0497), 12 dpi (*P* = 0.0243), 13 dpi (*P* = 0.0286) and 20 dpi (*P* = 0.0018) (**Figure 3**).

### 3.3 Impact of *V. ceranae* infection on SOD and PPO activities in *A. c. cerana* midguts of the workers

**Figure 4:**
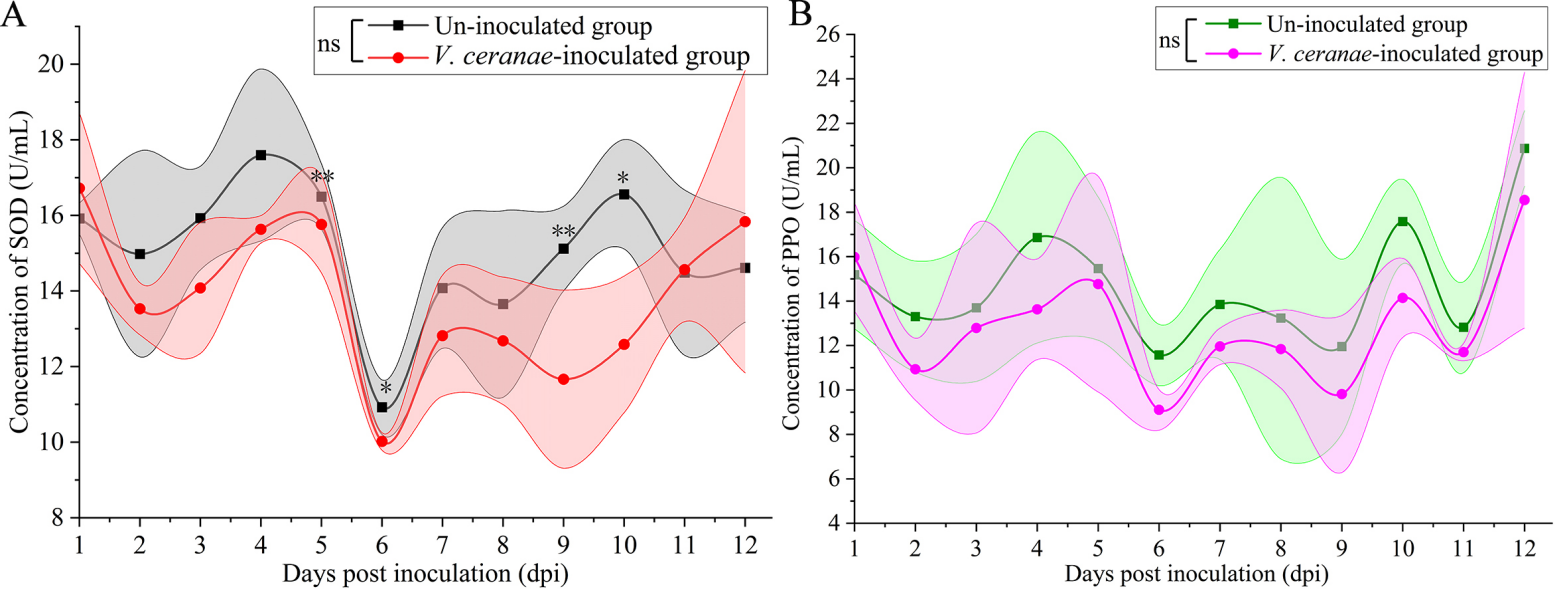
Detection of SOD **(A)** and PPO **(B)** activities in midguts of *V. ceranae*- and un-inoculated workers (*n*=3). Data were showed as mean ± SD, that analyzed using the Two-way ANOVA (Bonferroni’s *post-hoc* test), multiple t tests; ns indicates nonsignificance, * indicates *P* < 0.05, ** indicates *P* < 0.01.

Compared to that in the uninoculated group, the SOD activity in the *V. ceranae*-inoculated group was decreased at 2 (*P* = 0.2736)–10 dpi (*P* = 0.0399), with a significant difference observed at 5 dpi (*P* = 0.0097), 6 dpi (*P* = 0.0434), 9 dpi (*P* = 0.0015), and 10 dpi (**Figure 4A**). The PPO activity was consistently lower in the *V. ceranae*-inoculated group in comparison to that in the un-inoculated group at 2 (*P* = 0.2249)–12 (*P* = 0.5391) dpi, although no statistically significant difference was observed between these two groups (**Figure 4B**).

### 3.4 Impact of *V. ceranae* infection on CAT and GST activities in the midguts of *A. c. cerana* workers

**Figure 5:**
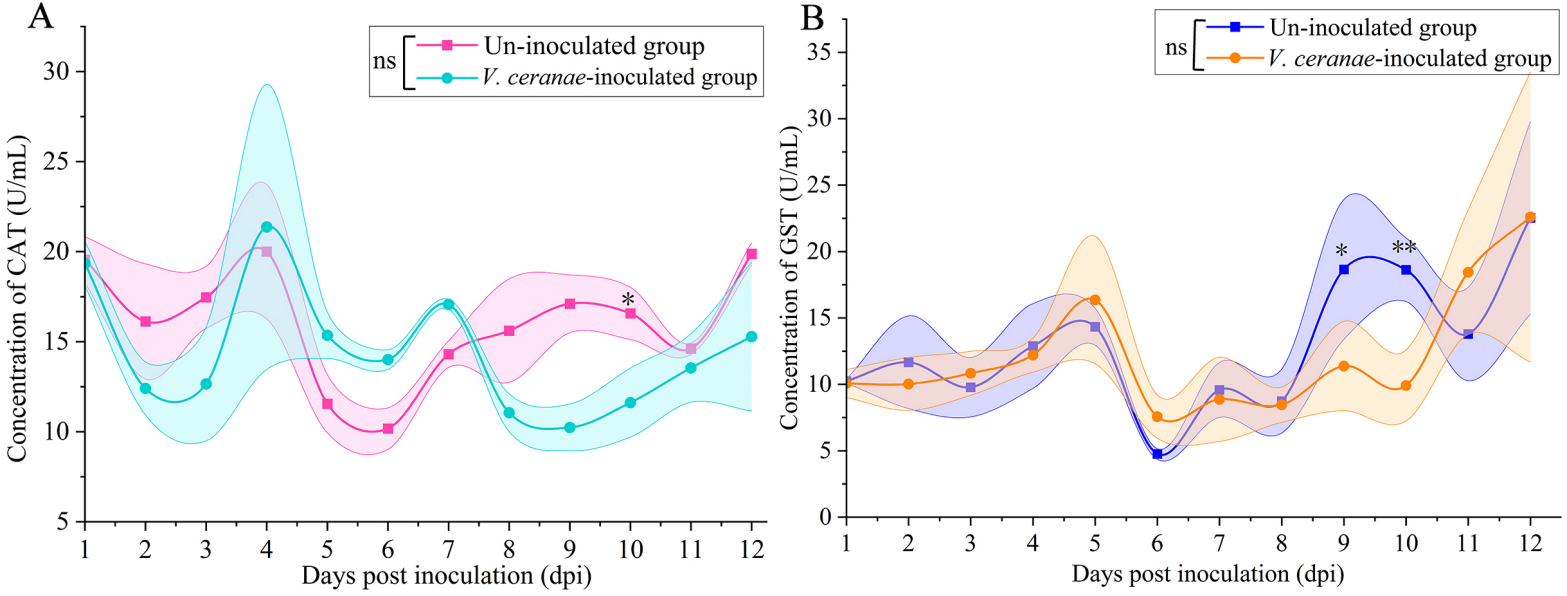
Detection of CAT **(A)** and GST **(B)** activities in midguts of *V. ceranae-* and un-inoculated workers (*n*=3). Data were showed as mean ± SD, that analyzed using the Two-way ANOVA (Bonferroni’s *post-hoc* test), multiple t tests; ns indicates nonsignificance, * indicates *P* < 0.05, ** indicates *P* < 0.01.

CAT activity was reduced in the *V. ceranae*-inoculated group in comparison with that in the un-inoculated group at 1 (*P* = 0.9209)–3 dpi (*P* = 0.0981), (**Figure 5A**); conversely, then was elevated in the *V. ceranae*-inoculated group at 4 (*P* = 0.1596)–7 (*P =* 0.1954) dpi; CAT activity was again downregulated in the host midgut following *V. ceranae* inoculation at 8 (*P* = 0.3183)–12 dpi (*P* = 0.4700), with a significant difference observed at 10 dpi (*P* = 0.0478) (**Figure 5A**). Additionally, GST activity was up-regulated at 5 (*P* = 0.5171)–6 dpi (*P* = 0.3692) and then reduced at 7 (*P* = 0.8196)–10 dpi (*P* = 0.0070), followed by an increase at 11 dpi (*P* = 0.1391), with a significant difference observed at 9 dpi (*P* = 0.0229), and 10 dpi (*P* = 0.0070) (**Figure 5B**).

### 3.5 Influence of *V. ceranae* infection on midgut epithelial cell structure of *A. c. cerana* workers

**Figure 6:**
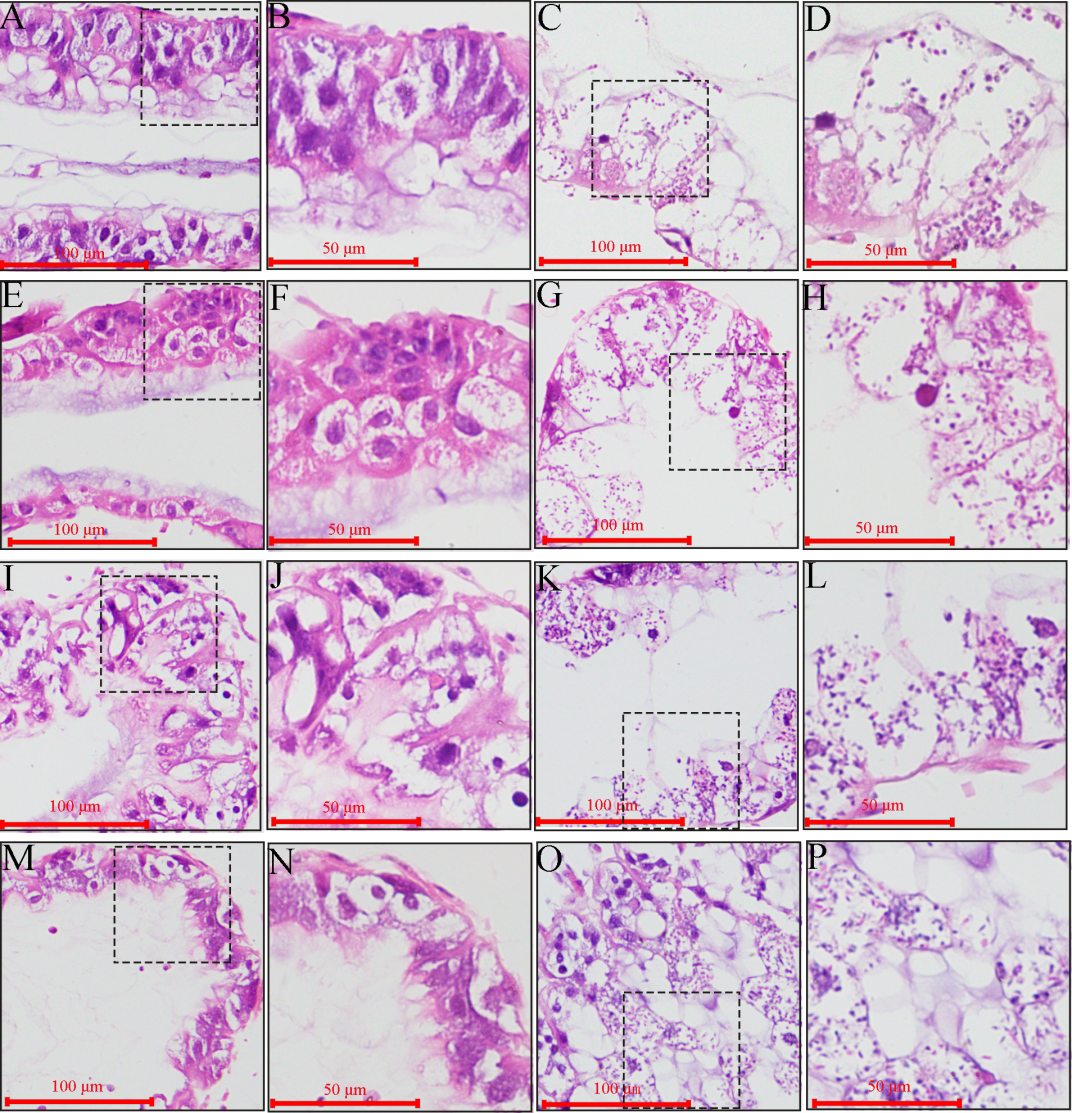
Microscopic observation of paraffin midgut sections of un-inoculated and *V. ceranae*-inoculated *A*. *c. cerana* workers. **(A, B)** Midgut tissue of un-inoculated worker at 7 dpi without spores; **(C, D)** Midgut tissue of inoculated worker at 7 dpi with *V. ceranae* spores; **(E, F)** Midgut tissue of un-inoculated worker at 8 dpi without spores; **(G, H)** Midgut tissue of inoculated worker at 8 dpi with *V. ceranae* spores; **(I, J)** Midgut tissue of un-inoculated worker at 9 dpi without spores; **(K, L)** Midgut tissue of inoculated worker at 9 dpi with *V. ceranae* spores; **(M, N)** Midgut tissue of un-inoculated worker at 10 dpi without spores; **(O, P)** Midgut tissue of inoculated worker at 10 dpi with *V. ceranae* spores. **(A, C, E, G, I, K, M, O)** were microscopic fields under 200× amplification, whereas **(B, D, F, H, J, N, P)** were microscopic fields under 400× amplification. Black dashed boxes show the selected region for observation under 200× amplification.

Microscopic observation of paraffin sections revealed darkly stained microsporidia in the midgut epithelial cells of *V. ceranae*-inoculated workers at 7–10 dpi (**Figures 6C, D, G, H, K, L O, P**), whereas no *V. ceranae* was detected in the midgut epithelial cells of uninoculated workers (**Figures 6A, B, E, F, I, J, M, N**). The number of *V. ceranae* spores in host cells gradually increased with increasing infection time (**Figures 6C, D, G, H, K, L O, P**).

In addition, the columnar and goblet cells were closely arranged, the epithelial cells were orderly arranged, the periesophageal membrane was clearly visible (**Figures 6A, B, E, F, I, J, M, N**), the outlines of columnar and goblet cells of *V. ceranae*-inoculated workers were arranged loosely and became unclear, and the periesophageal membrane was invisible (**Figures 6C, D, G, H, K, L O, P**). Moreover, as the infection progressed, the epithelial cells fell off the midgut wall and entered the intestinal lumen, together with the nuclei, cytoplasm, and various types of *V. ceranae* spores (**Figure 6**).

### 3.6 Influence of *V. ceranae* infection on lifespan of *A. c. cerana* workers

**Figure 7:**
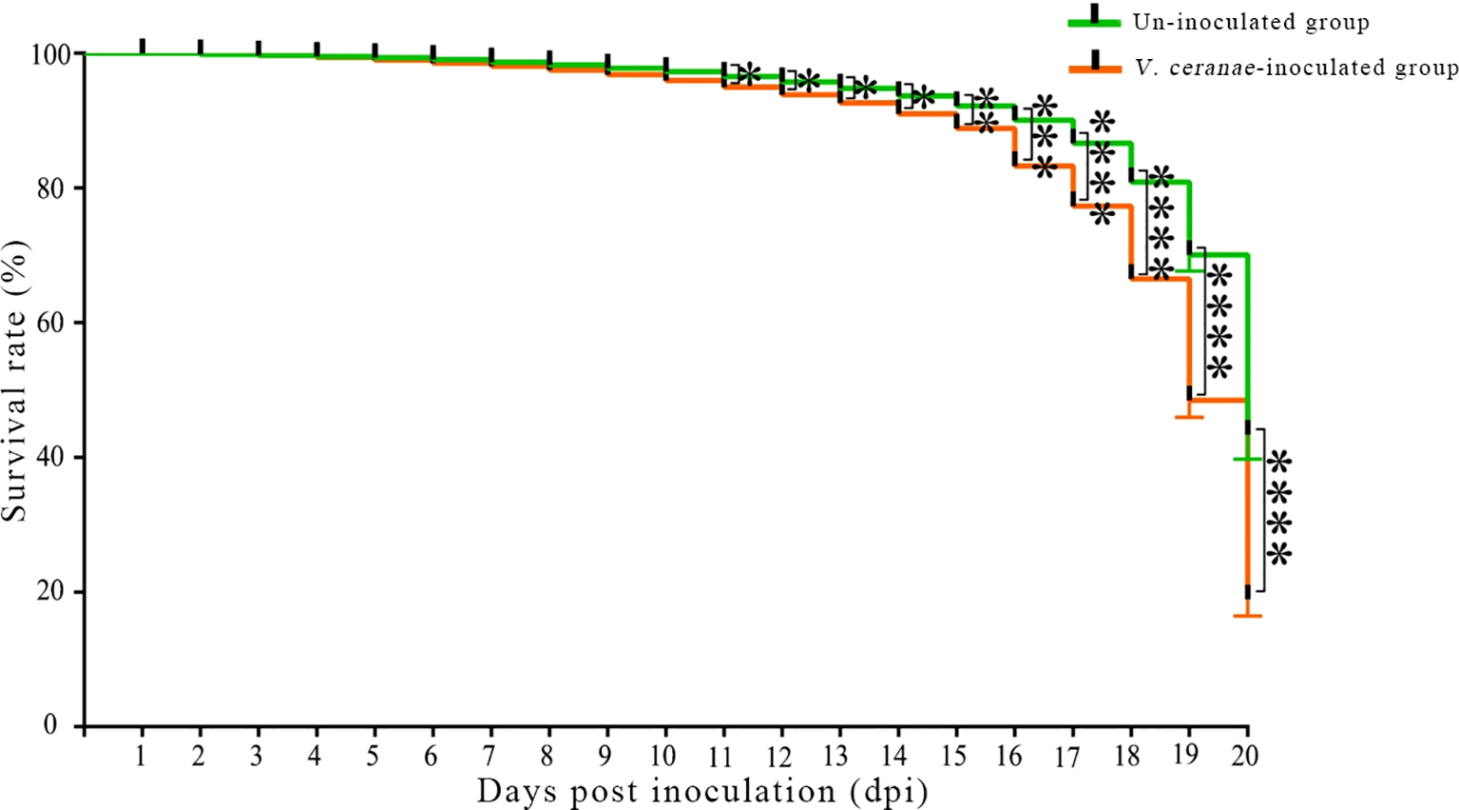
Survival rate of *A. c. cerana* workers after un-inoculation and inoculation with *V. ceranae* spores (*n*=3). Data were analyzed using the Log-rank (Mantel-Cox) test; * indicates *P* < 0.05, ** indicates *P* < 0.01, *** indicates *P* < 0.001, and **** indicates *P* < 0.0001.

The survival rate statistics indicated that the survival rates of workers in both the *V. ceranae*-inoculated and uninoculated groups at 1–5 dpi were high, and there was little difference between the groups (**Figure 7**). Survival rates in both groups began to decrease at 5 dpi (**Figure 7**). At 5–11 dpi, the survival rate of workers in the *V. ceranae*-inoculated group was lower than that in the un-inoculated group, whereas the survival rate of workers in the *V. ceranae*-inoculated group was significantly lower than that in the uninoculated group at 11–20 dpi (**Figure 7**).

## 4 Discussion

There is an ongoing debate and uncertainty surrounding the precise lifecycle duration of *V. ceranae* across different hosts. In *A. mellifera*, all intracellular stages of the life cycle of *V. ceranae* were observed at 3 dpi inside the worker’s epithelial cells using transmission electron microscopy, suggesting that the time for *V. ceranae* to complete the developmental cycle was 3 d (39). Based on other lines heterologous lepidopteran cell line IPL-LD-65Y *in vitro*, Gisder et al. reported that the life cycle of *N. apis*, the sister species of *V. ceranae*, is approximately 4 d (40). Although *A. cerana* is the original host of *V. ceranae*, its life cycle of *V. ceranae* in the midgut epithelium cells of *A. cerana* is unknown. Here, we observed a continuous increase in spore load from 1–12 dpi (**Figure 2**), indicative of sustained proliferation of *V. ceranae* within host cells. Additionally, the number of spores increased 10-fold every 4 d (**Figure 2**), suggesting that the life cycle of *V. ceranae* in the midgut epithelial cells of *A. cerana* workers was approximately 4 d, which differs from reports on *A. mellifera*. Intriguingly, we observed the presence of spores (3.5 ± 0.87 × 10^6^) as early as 1 dpi. Similar phenomenon was observed in previous studies, namely that by 3 h post inoculation, mature spores can be observed in the ventricular lumen of infected *A. mellifera* workers (39). We hypothesized that the detected *V. ceranae* spores at 1 dpi contained spores that did not infect the bee host cells or empty spores. However, further studies are needed to verify this hypothesis.

*Nosema* spp. have lost mitochondria during evolution, and thus must acquire energetic substrates directly from hosts for maintenance, growth, and reproduction (41, 42). Intestinal lesions caused by *V. ceranae* proliferation may decrease the absorptive capacity of honeybees and cause starvation symptoms, such as impoverishment of hypopharyngeal protein secretions (8). Chronic nutrient and energy stress suppress the innate immune system (43). After *V. ceranae* infection, workers of *A. mellifera* consumed a significantly higher amount of sucrose solution over the 24-h period tested (6). Here, we observed that the sucrose solution consumption of *V. ceranae*-inoculated workers was nearly always higher than that of uninoculated workers from 1–20 dpi, which indicated that the workers attempted to compensate for the imposed energy stress by consuming more sucrose solution. This is consistent with the results of a previous study on *A. mellifera* workers infected with *V. ceranae* (44). In summary, these results demonstrate that *V. ceranae* infection results in energy stress in both *A. mellifera* and *A. cerana* workers. In addition, the chronic stress caused by *V. ceranae* infection is likely to negatively influence foraging behavior, nutritional balance, immune response, and other important aspects of *A. cerana* workers, thus deserving more attention.

Insects maintain homeostasis in the body by neutralizing ROS and xenobiotics through the activation of various metabolic pathways that help protect cellular components from oxidative stress (26, 45). The antioxidant enzyme system, comprising SOD, PPO, CAT, and GST, is a critical physiological system for resisting oxidative stress in insects (46, 47). After infection with pathogens, the insect immune system stimulates the production of ROS, including superoxide radicals, to kill the invaders (24). CAT efficiently breaks down toxic hydrogen peroxide into harmless water and oxygen molecules (48). GST protects cells from oxidative stress by conjugating glutathione to ROS and other electrophilic compounds (49). It has been previously reported that GST is linked to oxidative stress response in the midgut of *Drosophila melanogaster* larvae (50). In the present study, CAT activity in the *V. ceranae*-inoculated group increased significantly at 5, 6, and 7 dpi (**Figure 5A**), whereas GST activity was significantly elevated at 6 dpi (**Figure 5B**). We inferred that the host enhanced the levels of CAT and GST activities in response to *V. ceranae*-caused oxidative stress in the middle stage of the infection process, thereby maintaining homeostasis of the internal environment. SOD catalyzes the dismutation of superoxide radicals into hydrogen peroxide and oxygen, thereby regulating ROS levels and preventing oxidative damage to cells (51). A significant decline in the activities of both SOD and GST was detected at 10 dpi, and CAT activity was reduced at both 9 and 10 dpi (**Figures 4A**, **5**). These results suggest that these three critical antioxidant enzymes are inhibited in the host midgut. This phenomenon may be attributed to the escalating spore load in the late infection stage, leading to a prolonged intensification of *V. ceranae* stress in the host and consequently suppressing the activities of SOD, CAT, and GST. PPO is involved in the melanization process, a common immune response in insects that involves the production of dark and pigmented structures that can trap and kill invading pathogens or parasites. In addition to its role in melanization, it simultaneously induces cellular and humoral immunity (52). Roberts and Hughes (53) found a negative correlation between PPO levels in the midguts of *A. mellifera* workers and *V. ceranae* infection intensity. Intriguingly, although the level of PPO activity in the *V. ceranae*-inoculated group was always lower than that in the uninoculated group, there was no significant difference between the groups (**Figure 4B**), indicating that PPO activity in the host midguts was not affected by fungal invasion. It is inferred that, as the host of origin of *V. ceranae*, *A. cerana* workers potentially evolved a strategy to counteract the negative influence of microsporidians on PPO activity. Further studies are required to explore these underlying mechanisms. Insects exhibit dynamic changes in GST and CAT in response to pathogen infection. For instance, in the larvae of Holotrichia parallela, following joint infection of entomopathogenic nematode and *Bacillus thuringiensis*, there was an initial increase in GST and CAT activity during the early stage, followed by a decrease in activity in the later stage (54). In this study, the contrasting alterations observed in GST and CAT levels between the middle and late stages of *V. ceranae* infection could potentially be ascribed to the insects’ initial augmentation of antioxidant enzyme activity as a mechanism of defense against the fungal invasion. Conversely, a decline in enzyme activity may occur in the later stage as a result of energy depletion or other physiological adjustments.

In a previous study, Higes et al. (39) detected that, 7 d after *V. ceranae* infection, the majority of epithelial cells showed evidence of degeneration, such as the presence of vacuoles in the cytoplasm or a brightly stained nucleus. Few unaltered cells were also observed, and either at the tips or at the bottom of the epithelial folds, there were cells containing *V. ceranae* intracellular stages. Here, we observed that the number of *V. ceranae* in the *A. cerana* worker midgut epithelial cells increased from 7 to 10 dpi (**Figure 6**), which is consistent with the findings of Higes et al. (39) Collectively, these results offer a solid basis for further investigations of *V. ceranae* infection in *A. cerana* workers, such as functional studies on fungal virulence factor-associated genes and the development of an RNAi-based control strategy.

*V. ceranae* infection damaged the structure of midgut epithelial cells in *A. mellifera* workers (7). Based on microscopic observations by optical microscopy and transmission electron microscopy, Fries et al. (2) first reported that *V. ceranae*-infected ventriculus cells of *A. cerana* workers were shed into or burst open into the gut lumen where mature spores were released, and described the size of the spores. Darkly stained microsporidia were clearly detected in the midgut epithelial cells of *V. ceranae*-inoculated workers at 7–10 dpi (**Figure 6**). Additionally, the boundaries of the midgut epithelial cells in *V. ceranae*-inoculated workers were blurred, and the nuclei almost disappeared, with nucleic acid substances dispersed around the cytoplasm (**Figure 6**). These results suggest that *V. ceranae* infection also causes serious damage to the structure of the midgut epithelial cells of *A. c. cerana* workers. Collectively, the findings from previous studies (2) and this study offer experimental evidence for the damage caused by *V. ceranae* infection to the structure of *A. c. cerana* worker midgut epithelial cells, laying the foundation for further investigation of the pathogenesis of bee nosemosis.

Paris et al. (55) assessed the survival rate and eating behavior of *A. mellifera* workers infected with *V. ceranae* for 1–22 d, and the results showed that the survival rate of infected workers was significantly lower than that of uninfected workers. We previously observed that the mortality rate of *A. mellifera* workers inoculated with *V. ceranae* spores gradually increased over time, and that the mortality rate of *V. ceranae*-inoculated workers was significantly higher than that of uninoculated workers at 7 and 10 dpi (33). It has been documented that *V. ceranae* infection significantly increased the mortality rate of *A. cerana* workers (56). In this study, the survival rates of workers in both the *V. ceranae*- and un-inoculated groups were high at 1–5 dpi (**Figure 7**), implying that *V. ceranae* had little influence on host longevity at the early stage of infection. However, at 5–20 dpi (**Figure 7**), the survival rate of *V. ceranae*-inoculated workers was always lower than that of uninoculated workers, and the difference was significant at 11–20 dpi, indicating that *V. ceranae* infection shortened the lifespan of *A. cerana* workers, similar to the findings of Huang et al. (56). In conclusion, *V. ceranae* can negatively affect the midgut epithelial cell structure and lifespan of *A. c. cerana* workers during infection.

**Figure 8:**
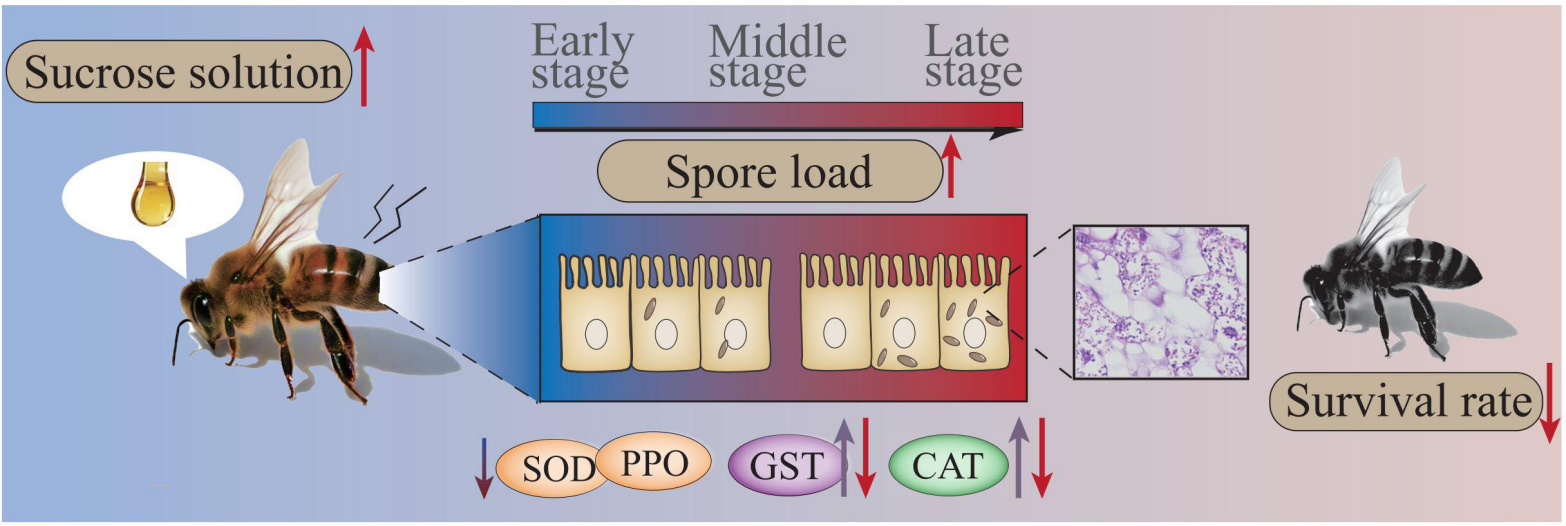
Hypothetical schematic diagram of the impact of *V. ceranae* infection on *A. c. cerana* worker.

Collectively, the findings of the present study indicate that the spore load continuously increases as *V. ceranae* proliferates in the midgut of *A. c. cerana* workers, giving rise to continuous energetic stress for the host, apparent damage to the host midgut epithelial cell structure, induction of CAT and GST activities in the host midgut at the middle stage, and inhibition of the activities of SOD, CAT, and GST at the late stage of infection, which ultimately shortens the host lifespan (**Figure 8**).

## Data Availability

The original contributions presented in the study are included in the article/supplementary material. Further inquiries can be directed to the corresponding author.
